# LACE 2.0: an interactive R tool for the inference and visualization of longitudinal cancer evolution

**DOI:** 10.1186/s12859-023-05221-3

**Published:** 2023-03-17

**Authors:** Gianluca Ascolani, Fabrizio Angaroni, Davide Maspero, Francesco Craighero, Narra Lakshmi Sai Bhavesh, Rocco Piazza, Chiara Damiani, Daniele Ramazzotti, Marco Antoniotti, Alex Graudenzi

**Affiliations:** 1 Department of Informatics, Systems and Communication, University of Milan-Bicocca, Milan, Italy; 2 Department of Biological Sciences, Birla Institute of Technology and Science (BITS), Pilani, Rajasthan India; 3 Department of Medicine and Surgery, University of Milan-Bicocca, University of Milan-Bicocca, Milan, Italy; 4 Department of Biotechnology and Biosciences, University of Milan-Bicocca, Milan, Italy; 5 Bicocca Bioinformatics Biostatistics and Bioimaging Centre - B4, University of Milan-Bicocca, Vedano al Lambro, Monza, Italy

**Keywords:** Single cell, Longitudinal, Clonal analysis, Shiny, Interface

## Abstract

**Background:**

Longitudinal single-cell sequencing experiments of patient-derived models are increasingly employed to investigate cancer evolution. In this context, robust computational methods are needed to properly exploit the mutational profiles of single cells generated via variant calling, in order to reconstruct the evolutionary history of a tumor and characterize the impact of therapeutic strategies, such as the administration of drugs. To this end, we have recently developed the LACE framework for the Longitudinal Analysis of Cancer Evolution.

**Results:**

The LACE 2.0 release aimed at inferring longitudinal clonal trees enhances the original framework with new key functionalities: an improved data management for preprocessing of standard variant calling data, a reworked inference engine, and direct connection to public databases.

**Conclusions:**

All of this is accessible through a new and interactive Shiny R graphical interface offering the possibility to apply filters helpful in discriminating relevant or potential driver mutations, set up inferential parameters, and visualize the results. The software is available at: github.com/BIMIB-DISCo/LACE.

## Background

We present LACE 2.0, a new R package to analyze single-cell (SC) mutational profiles generated from *longitudinal* sequencing experiments of cancer samples, which are increasingly becoming available due to the continuous advances in biotechnology. Robust computational frameworks for SC sequencing data are needed to mitigate the impact of technology-related errors, as well as that of sampling limitations, so as to deliver a fine characterization of intra-tumor heterogeneity and to possibly cast a light on the mechanisms underlying drug resistance and relapse under a variety of conditions and experimental setups. In recent years, excellent tools for the inference of clonal/mutational trees for SC experiments, but incapable of handling longitudinal data [1, 2], or longitudinal bulk data[3] have been proposed to investigate the mutational evolution of cancer. LACE 2.0 embraces the possibility to analyze the development of the disease using SC data from longitudinal studies and is capable of deriving a clonal tree tagged with time coordinates coherent with the sampling temporal order.

In this context, the original version of LACE was designed to exploit binarized mutational profiles, generated via the application of standard pipelines for variant calling to either DNA- or (full-length) RNA- single-cell sequencing experiments, performed at multiple time points. To do so, LACE maximizes a weighted likelihood function by solving a Boolean matrix factorization problem via MCMC sampling [4], and proved to be accurate and robust even with high rates of noise and data-specific errors.

Compared to the former release of LACE, this version introduces the possibility to use standard VCF and BAM formats as input of the clonal analyses. Previously, the whole pipeline for retrieving putative driver mutations and producing the phylogenetic matrix, or performing all sanity checks including time order logic to avoid spurious longitudinal clonal tree, was left to the discretion of the users.

The latest LACE version (2.0) extends and enhances the original version in several ways. Thanks to a completely new Shiny app interface [5], LACE 2.0 includes: (a) an easy-to-use module for data preprocessing and organization, which allows one to test and adjust quality and relevance filters, and to annotate gene variants; (b) a module for the interactive visualization of the inferred cancer evolution models, which returns both the *longitudinal clonal tree* and the *fishplot*, and allows one to inspect the model results (i.e., clones, temporal relations and prevalences), as well as to query the external database Ensembl for further information [6]. Importantly, LACE 2.0 requires a limited set of parameters, which are sufficient to mitigate the source of noise and infer reliable models of cancer evolution (see [4] and [7] for further details).

## Implementation

LACE 2.0 exploits binarized mutational profiles (1 if a mutation is present in a given cell, 0 if it is absent, NA for low coverage) generated by calling variants from SC DNA- or full-length RNA-seq experiments of cancer samples collected at multiple time points, usually before and after therapy. LACE 2.0 returns as output a *longitudinal clonal tree* [4], in which nodes represent *clones*, i.e., groups of single cells with the same genotype, and edges represent either *parental relations*, e.g., clone B originates from clone A thanks to the acquisition of a new mutation, or *persistence relations*, e.g., clone C is present at both time points 1 and 2.

LACE 2.0 is built as a shiny app allowing the user to apply filters, perform operations on the data and visualize the results.

The interface is organized in 8 modules represented by tabs:

- Project Creation
- Metadata Info
- Gene Variant Annotations
- Quality Filters
- Single Cell Sampling Depth
- Variant Filters
- Inference
- Longitudinal Display

Stepping through each of these modules one can select mutations according to distinct criteria in order to generate the input for the inferential routines, set parameters for the derivation of the longitudinal relations among clones and display the most likely clonal tree and hyper parameters describing the time evolution of the samples, see Table 1.

### Inputs

LACE 2.0 requires as input the result of standard alignment and variant calling pipelines, applied to either SC DNA-seq or (full-length) RNA-seq data. Concerning the former, even though whole-genome and whole-exome SC sequencing experiments may be appropriate for genotyping, they are rarely available due to the high costs, so targeted genome sequencing represents a more appropriate option to this end. Also full-length SC RNA-sequencing experiments (e.g., SmartSeq) can be employed in LACE 2.0, despite the known issues related to coverage, which is typically low and limited to transcribed regions [7]. Yet, this data type is extremely widespread and has the notable advantage of providing a natural mapping between the genotype and the gene expression of single cells. The first preprocessing step (*not* included in LACE 2.0) consists in aligning the library to a reference genome and performing variant calling. This can be done using any of the widely used pipelines (see [8]). Using LACE 2.0, single variants can be then annotated using Annovar [9] as a back-end. LACE 2.0 takes as input the BAM and the VCF files, and allows the user to set different quality and relevance filters with a simple interactive form of the Shiny interface.

### Project creation

**Fig. 1:**
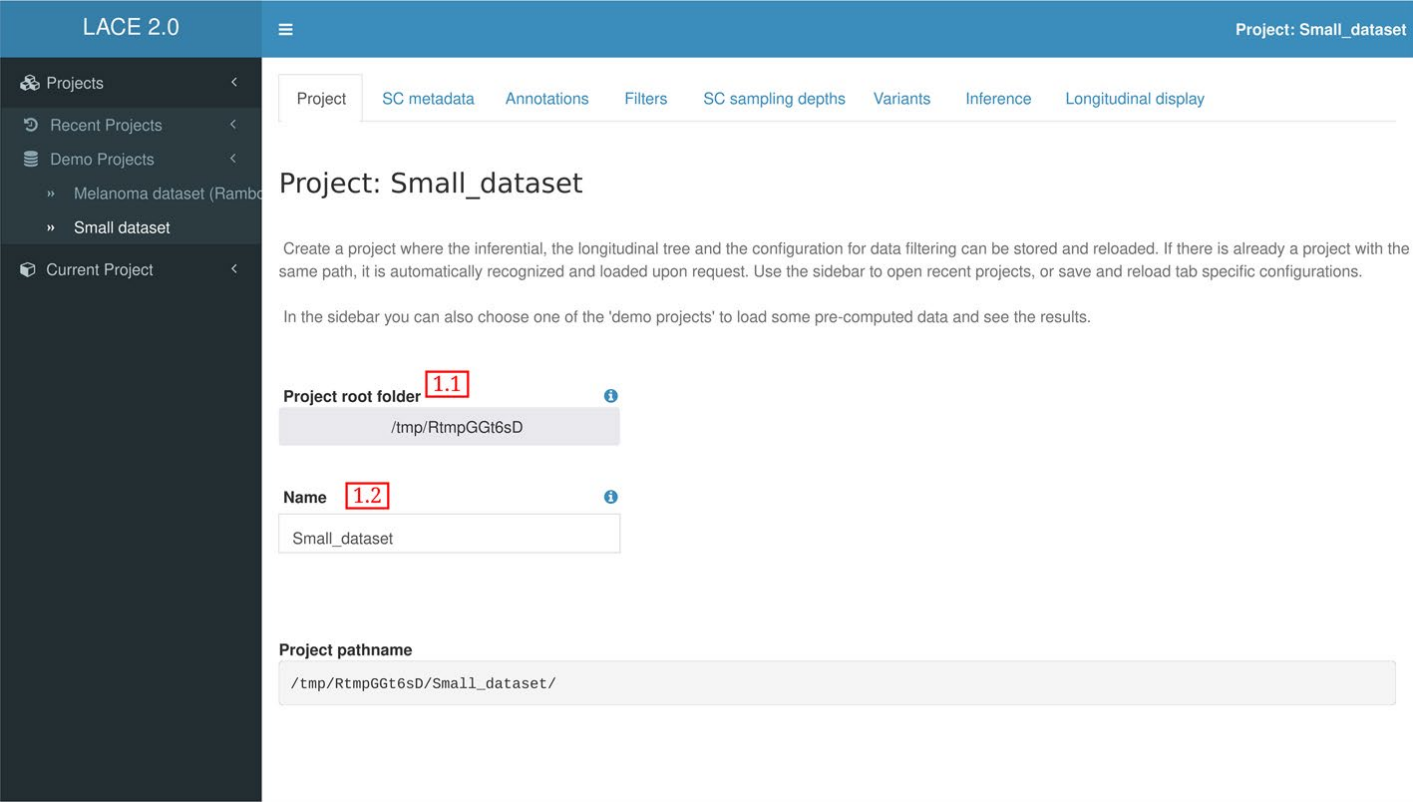
LACE 2.0 Project. LACE 2.0 allows to create new or load former and recent opened projects

In order to organize analyses with different parameters and to store intermediary steps, each inferential experiment with all the setting are saved in a subfolder named after the project id which is selected by the user (Fig. 1 field 1.1 and field 1.2). At the moment a project is created, default configuration settings are generated marking the subfolder as part of the LACE 2.0 analysis. If the selected subfolder is already a LACE 2.0 project, the app recognizes it and loads with the last configuration used. A project is saved after each user interaction with the interface, and it can be reloaded directly by selecting the project folder or by the sidebar where the latest project names are displayed.

### Metadata info

**Fig. 2:**
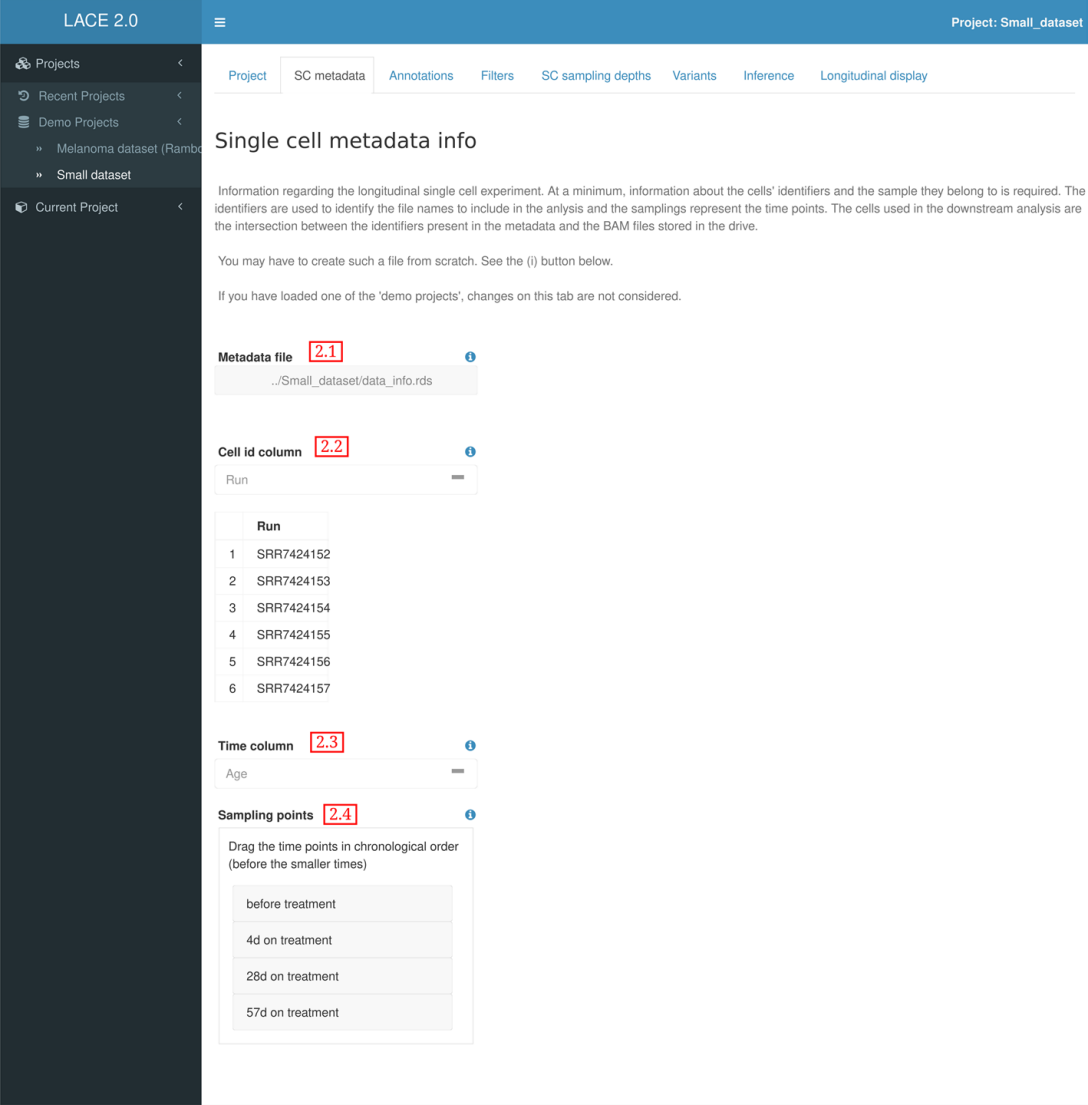
LACE 2.0 Metadata Info. Select the SC id and the experiments’ time coordinate and order

In order to perform clonal analyses, it is required to provide information relative to the experiment such as the cell IDs which are used to retrieve the VCF/BAM files and the sample name each cell belongs to (Fig. 2 field 2.1). This metadata is generally included with the experimental sequencing data as part of the library preparation. LACE 2.0 accepts tsv/csv tabular formats with headers and rds files containing standard tabular data R format (convertible to a dataframe type). The metadata file can include more information relative to the experimental setup and the platform used.

After selecting the single cell ID (Fig. 2 field 2.2) and the sample name columns (Fig. 2 field 2.3), the user can reorder the samples in chronological order Fig. 2 (field 2.4). This is the only step where time information is provided during the analysis, and it will be used in post inferential computation to generate the longitudinal clonal tree and the ordered sets of clonal prevalences.

### Gene variant annotations

Under the infinite sites assumption (ISA), clonal analysis is performed by grouping cells in clades with sets of similar mutations and by linking them via inclusion relation of mutation sets. In order to avoid excessive partition of cells over variants with unspecified roles in the phenotypical cascade, as in diseases with high mutational rate, it is useful to annotate mutations so as to select only relevant classes. LACE 2.0 performs annotations of variant calling data using Annovar as back-end. Each mutation of each cell is annotated based on the annotation database used. Annovar provides a database for the human species useful for tagging cancer mutations and, whenever possible, providing their functional effect.

If Annovar’s Perl scripts are available in the OS standard binary paths, LACE 2.0 automatically detects them and sets their path; otherwise, the user should provide the folder containing the Perl scripts. The user should also provide the database to use for the annotation and the folder containing the variant calling files in VCF format obtained by standard pipelines.

### Quality filters

All types of SC sequencing data is characterized by various sources of noise which depends on the technology used. Detected mutation might be characterized by low quality score or low statistical power. Some mutations can be neglected, while others might cause relevant effects, especially on exonic regions where variations can result in changes of the translational process and modify their functional form.

**Fig. 3:**
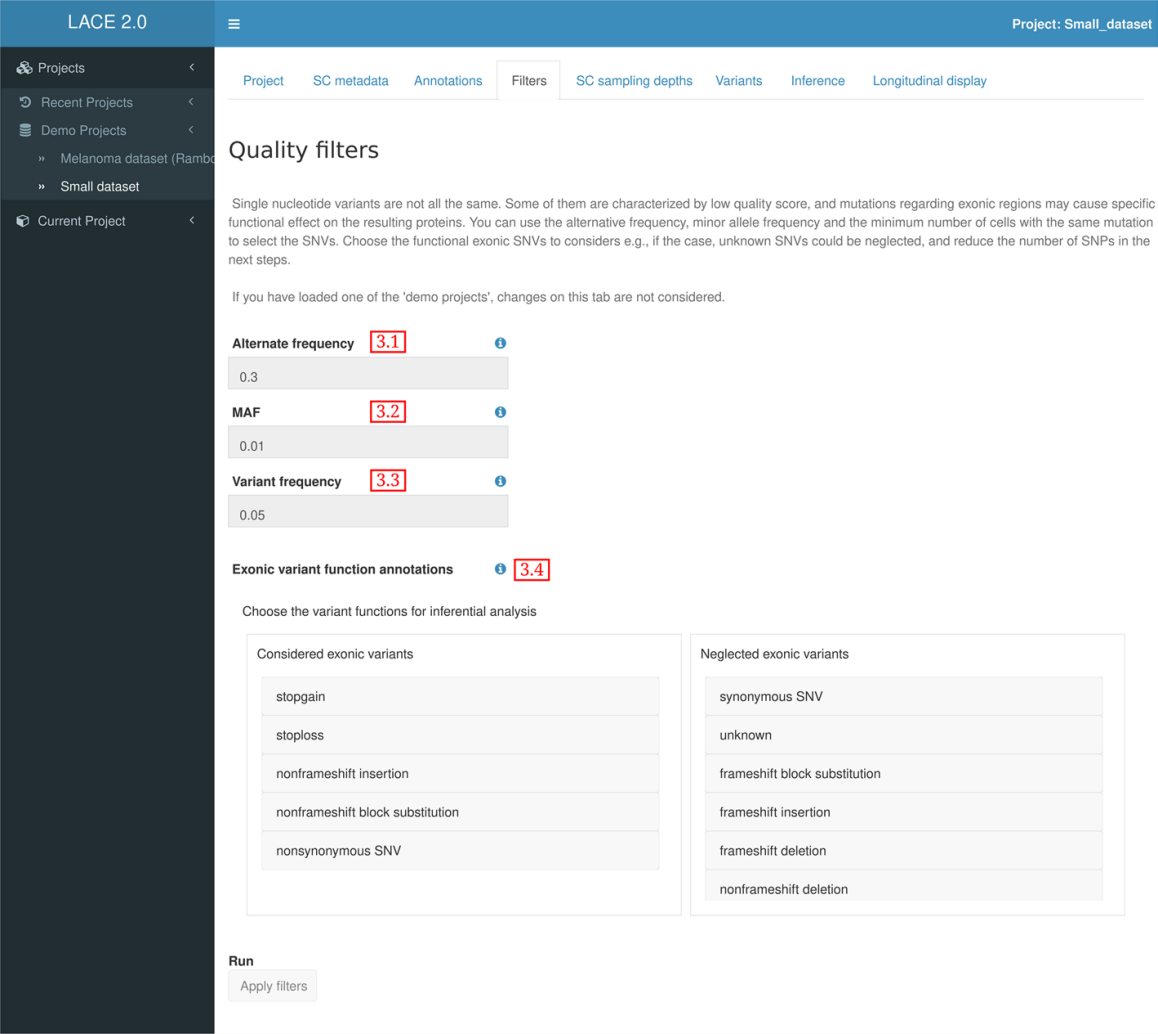
LACE 2.0 Quality Filters. Mutation selection can be performed by setting thresholds for read variant based evidences and by selecting mutations with specific exonic functions

In order to avoid small and possibly spurious fluctuations on sequencing data, variants can be filtered if they have low supporting evidences (Fig. 3). The user can set the minimum values for:

- The number of reads supporting the alternative alleles in a cell. Due to sampling noise, cells not showing enough alternate reads for a mutation are considered not mutated at that site and are not counted (Fig. 3 field 3.1).
- The frequency of the minor allele for each referenced SNP included in a default global population. To assess the significance of somatic mutations, if variants are absent in control subjects or the variants have very low MAF (e.g., $< 0.05$), they are marked as somatic mutations. In comparison, if the MAF in the general population is big enough ( e.g. $> 0.01$), they are considered possible deemed polymorphic or benign variants [10–12] (Fig. 3 field 3.2).
- The cell frequency per sample showing the mutation at the same site. Mutations which are not supported by a minimum amount of cells at each sample can be excluded, if necessary, due to heterogeneity and fluctuations (Fig. 3 field 3.3).

Furthermore, the user can choose which functional exonic variation should be considered or neglected. For example, there are cases in which unknown and synonymous mutations in exonic regions are disregarded because their effect cannot be explicitly related to the experimental condition. All possible variant functions and their explanations are resumed in Table 2.

At the end of this module, a time-indexed binarized matrix representing the remaining mutations for each the cell in each sample is generated. For any given cell, SNVs exceeding the number of reads supporting the alternative alleles threshold will be set to 1, or 0 otherwise.

### Single cell sampling depth

The number of reads per variant site represents an optimal filter to retrieve relevant mutations with sufficient supporting evidence in the signal. Read depths along the sequences are usually not provided in standard alignment or variant calling pipelines. The user should provide the Samtools Suite [13] executable folder location in case LACE 2.0 cannot find back-end in the OS standard folder. Furthermore, the folder with the longitudinal single cells aligned data should be provided as input for the computation. To be noted, aligned files must have a case insensitive “.bam” extension, and only filenames coinciding with the cell IDs provided in the metadata info are analyzed, while other files are neglected. Due to the fact that retrieving depth for all SC at variant sites is computationally expensive, the procedure is only performed on sites passing the quality filters previously set.

### Task manager

LACE 2.0 tries to limit time consuming steps using a task manager based on file timestamps specifically developed for the purpose of reducing unnecessary heavy computations. More precisely, a computation step is defined by the input files, the output files and the required parameters stored in a temporary configuration file. If a task is a bijective function, the task manager checks if input file timestamp < configuration file timestamp < output file timestamp, and only for those files for which the relations do not hold true, the task is recomputed. If a task is an injective or a surjective function, the task manager controls if all input file timestamps < configuration file timestamp < all output file timestamps, and the task is recomputed only if the relations do not hold true.

This is very useful when tasks are chained, and the user modified parameters of downstream steps or if the user decided to add new acquired samples in the analysis (e.g., annotation and depth at variant sites can require a long computational time).

### Variant filters

**Fig. 4:**
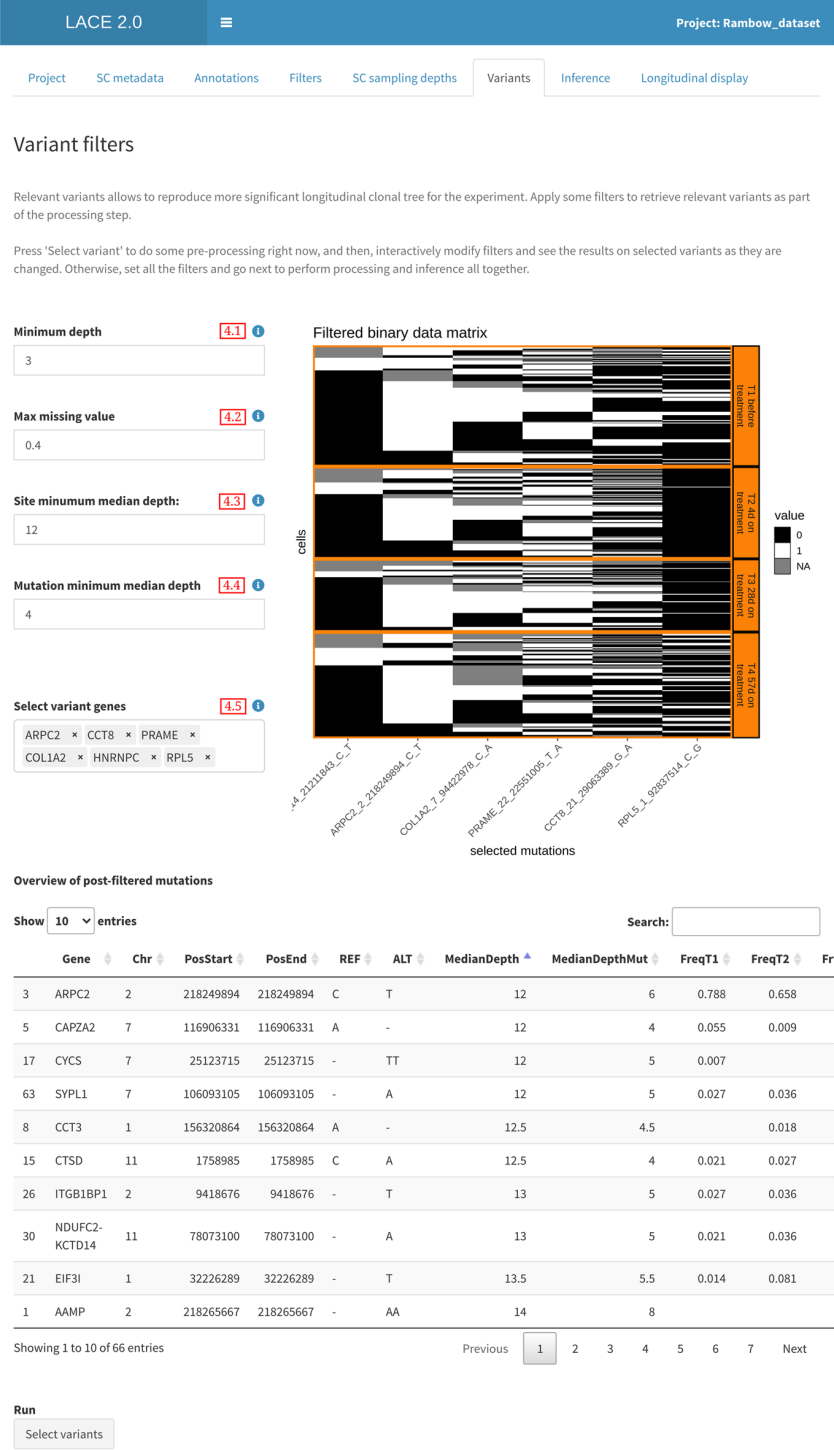
LACE 2.0 Variant Filters. Relevant and driver variants among all the sample mutations can be selected by setting read depth filters per mutation site and interactively completed by the user

Not all mutations are distinctive of the disease or experiment under study. Identifying relevant and driver variants allows to reproduce a more significant longitudinal clonal tree. The user can select a set of filters based on gold standards and other analyses such as:

- The minimum number of reads at given mutation site. Depth represents the strength of the signal measured, and this filter marks previously identified mutations based on read depth at the single cell level. Where the signal falls below a given threshold, the corresponding mutations in a cell are less reliable and set to ’NA’ (Fig. 4 field 4.1).
- The maximum number of missing data per gene. If a mutation is marked as ’NA’ in too many cells, the same knowledge on the supposed mutation site becomes more undefined; hence, it is prone to be marginal (Fig. 4 field 4.2).
- The minimum median depth per locus. This filter ensures the site is sufficiently measured given the set of experiments sampled (Fig. 4 field 4.3).
- The minimum median depth supporting the mutation. Similarly, the signal supporting the mutation should be sufficiently measured in the whole set of cells included in the analysis (Fig. 4 field 4.4).
- Subset of known genes. If there are genes associated with treatments or are drivers for the disease or relevant for the understudied longitudinal experiment, they can be included directly by the user. The proposed list of gene names are obtained from the list of mutated genes which have passed the applied filters (Fig. 4 field 4.5).

The final set of variant sites passing all filters are used to subset the binarized matrix obtained in the former module (Fig. 4). Furthermore, values in the matrix corresponding to mutations falling in a position with a coverage below the above threshold are set to ’NA’. This produces a time-indexed binarized mutational matrix, which is processed by the inference engine.

Finding the parameters to select variants is not an easy task, and the user might not know in advance how to choose the best set of filters. Hence, the user can apply the aforementioned filters and computations on VCF and BAM files to derive all the necessary aggregated information on the sampled cells. Afterward, the user is presented with an interactive live preview of variants passing the filters (including relevant parameters which can help in the selection) while values are changed.

### Inference

**Fig. 5:**
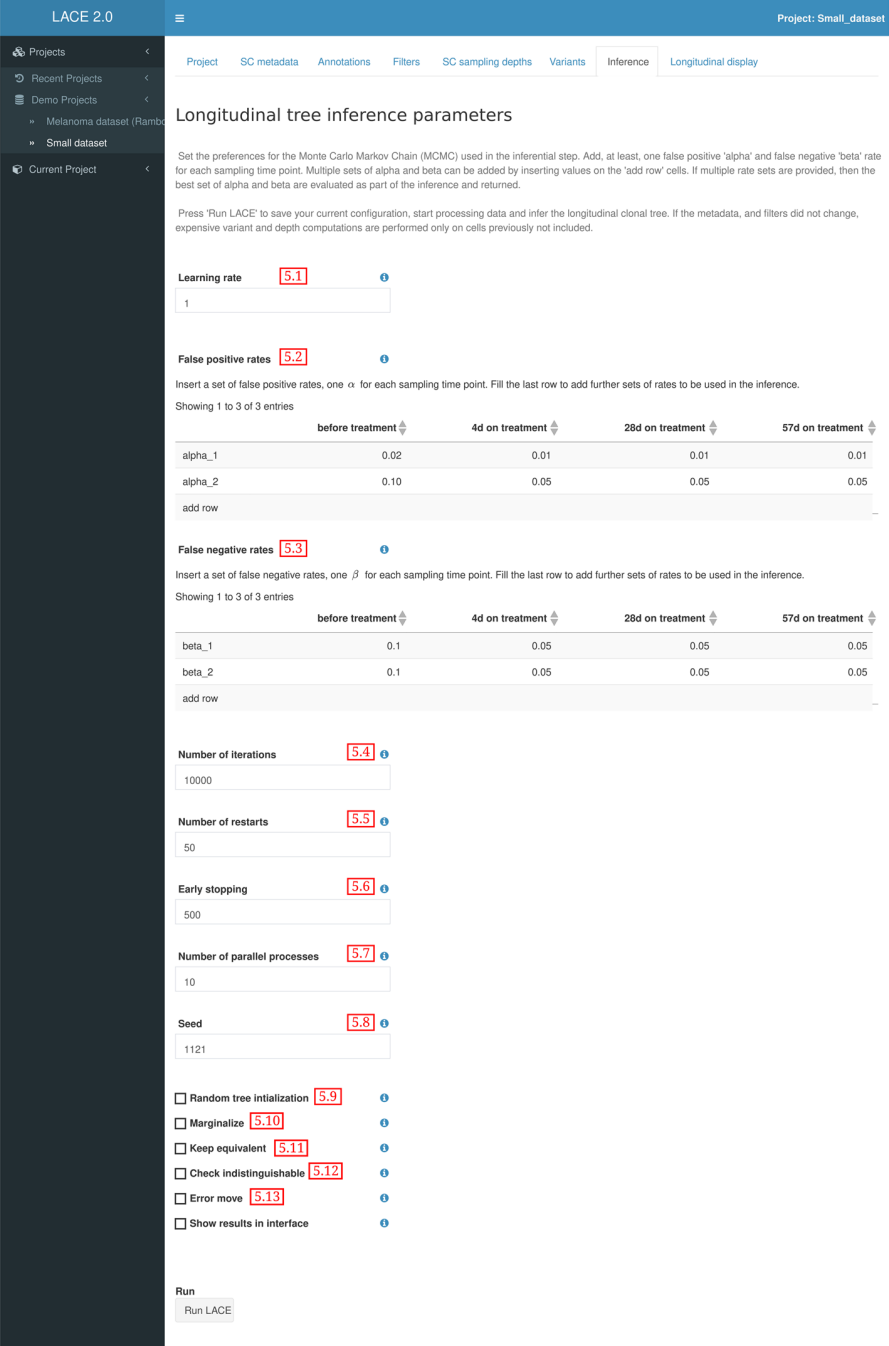
LACE 2.0 Inference. Set of parameters to perform longitudinal inference of mutational events. It is possible to set the learning rate, number of iterations, the number of false or negative rates of each experiment included in the project and more specific parameters to perform the MCMC inference

The inferential tab allows the user to set all the parameters to solve the boolean matrix factorization problem and estimate the model parameters of the model using a MCMC to maximize the likelihood (Fig. 5). The inferential step uses the following set of parameters:

- Learning rate (Fig. 5 field 5.1)
- False positive rates for each sample (Fig. 5 field 5.2)
- False negative rates for each sample (Fig. 5 field 5.3)
- Number of iterations in each MCMC search (Fig. 5 field 5.4)
- Number of restart for the MCMC (Fig. 5 field 5.5)
- Early stopping number of iterations with no growing likelihood (Fig. 5 field 5.6)
- Number of parallel processes (Fig. 5 field 5.7)
- Random seed to recreate simulations (Fig. 5 field 5.8)
- Initialize the clonal tree randomly (Fig. 5 field 5.9)
- Marginalize the cell attachment matrix (Fig. 5 field 5.10)
- Keep equivalent solutions and return all of them (Fig. 5 field 5.11)
- Check indistinguishable event and remove them (Fig. 5 field 5.12)
- Estimate error rates of MCMC moves (Fig. 5 field 5.12)

The user can insert more than one false positive rate and one false negative rate value for each sample. During the inferential step, the maximization of the likelihood for each set of rates is performed, and the best results are returned.

### Longitudinal clonal tree

The model adopted in LACE 2.0 (see also [4]) describes the clonal factorization of the underling evolutionary process based on experiments measured at sequential time points under the ISA assumption and subjected to different sources of noise derived from sampling single cells sequencing data.

The model can be explained by the following matrices:

- the single cell data matrix *D* where rows represent single cells, columns are all the mutations considered and entries are 1 when mutations are present and zero when absent,
- the cell attachment matrix *C*, where rows identify the single cells, columns are the clones and values are 1 for cells belonging to a clone,
- the phylogenetic matrix *B*, where rows are the clones, columns are the mutations, and it is a boolean triangular matrix equivalent to a tree with entries associating clones to mutations.

These matrices can be related via tensorial product by the following relation:

$$\begin{aligned} D=C \cdot B \end{aligned}$$

which describes the model as a boolean factorization problem with constraints. The real sequenced data matrix *G* has the same shape of *D* and represents similar relations between sampled cells and measured variants such that values are 1 when a mutation is observed, zero when not or NA if there are no sufficient reads supporting the observations. Discrepancies between the optimal solution of the model describing the underlying experiment $\tilde{D}$ and real data *G*,

$$\begin{aligned} G\approx \tilde{D}, \end{aligned}$$

are characterized by depth and sequencing tech limitations used to measure each sample *s* at time $t_s$ which generate false event occurrences with platform dependent false positive $\alpha _s$ and false negative $\beta _s$ rates. Therefore, the Bayesian approach is determined by:

$$\begin{aligned} P(B,C|G)\propto P(G|B,C) P(B,C), \end{aligned}$$

and the maximization of the likelihood with constraints on the arguments *B*, *C* is given by:

$$\begin{aligned} P(G|B,C) = P(G|\tilde{D}). \end{aligned}$$

The model assumes that the longitudinal experiments are sampled from a unique generative phylogenetic matrix *B*; consequently, without further time and cellular cooperation constraints, the likelihood with sample (platform) specific weights can be completely factorized by mutation and by single cell, rendering the model time agnostic and tractable:

$$\begin{aligned} P(G|B,C)=\prod _s \left[ \prod ^{n_s}_{i=1}\prod ^m_{j=1} P(G_{i,j} |\tilde{D}_{i,j} )\right] ^{w_s}, \end{aligned}$$

where *s* is the sample, $n_s$ is the number of cells in *s*, $w_s$ is the sample specific weight depending on the number of cells in the sample and *m* is the index of the total set of mutations considered.

The clonal tree *B* and attachment matrix *C* maximizing the weighted likelihood function is computed on all time points, via a MCMC search scheme.

For each clonal matrix *B* exists one and only one clonal tree. The construction of the clonal tree from the clonal matrix *B* is given by the following set of operations:

1. a node of the clonal tree is generated for each row of *B*,
2. a clone *i* is characterized by the acquired mutations which are the ones in the *i*-th row of *B*, $B_i$,
3. the root of the clonal tree is the row *k* for which $||B_k||_1=1$ holds true,
4. given a clone *i*, its parent *j* among all the clones *k* with $||B_k||_1< ||B_i||_1$ is given by $j={{\,\mathrm{arg\,min}\,}}_k\left( ||B_i-B_k||_1\right)$.

The last point is a direct consequence of the ISA assumption which excludes the possibility of back mutations.

A longitudinal clonal tree is an augmented clonal tree where nodes are duplicated for each sampling time $t_s$ since the first time a clone has been observed, are labeled with increasing time tags going from $t_s$ to the final experimental sampling time and same clones with consecutive times are joined.

Reproducing a longitudinal clonal tree from *B* is not a straight forward procedure. First, it should be noticed that observing a clone implies the usage of the knowledge coming from the clonal prevalence *C*. Second, if clones *i* is parent of clone *j*, than there is no guarantee the first observation time $t_v$ of clone *i* and the first observation time $t_w$ of clone *j* satisfy the relation $t_v \le t_w$.

To avoid spurious appearance of child clones before parent clones, LACE 2.0 checks the time tags of the longitudinal clonal tree with the order of the sampling times. In case of time reversal among two time tagged nodes, it adds a node to the longitudinal tree representing the parent clone with prevalence zero and with the smallest time tag of the two considered nodes. Compared to LACE, LACE 2.0 returns the augmented longitudinal clonal tree with time tagged nodes corresponding to the measured sequential time points of the experiment without time reversal among parent–child nodes.

### Longitudinal display

**Fig. 6:**
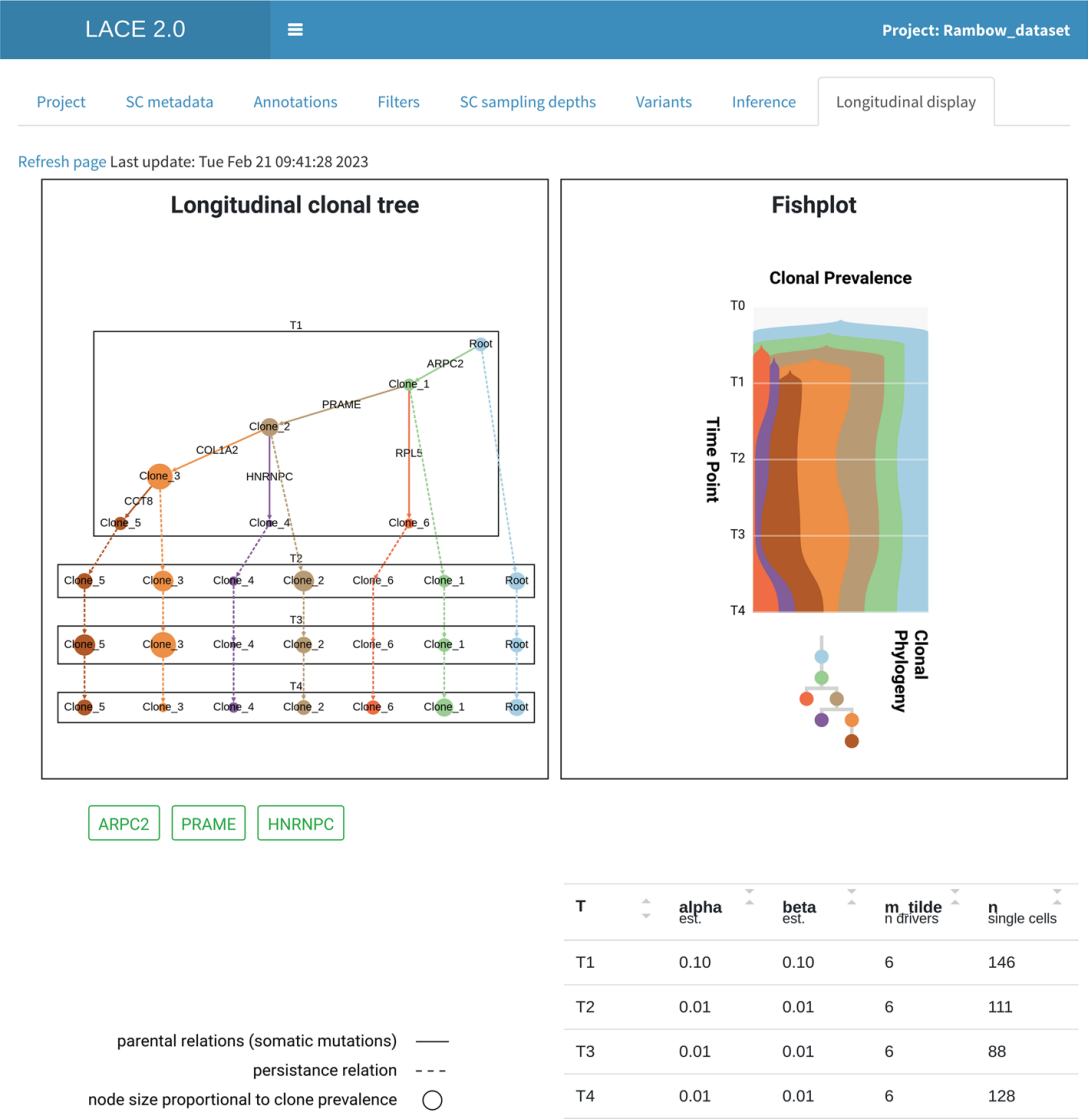
LACE 2.0 output. (Left) The longitudinal clonal tree of the BRAF-inhibitor treated melanoma PDX described in [16] is displayed. Nodes represent clones and edges either persistence or parental relations, and node size is proportional to clonal prevalence. (Right) LACE 2.0 also returns the fishplot, in addition to the summary table. The Shiny interface is fully interactive with each element reactive and the possibility of obtaining information from external databases such as Ensembl [6]

After the inference, the Shiny interface of LACE 2.0 allows one to visualize and interact with the output cancer evolution model (Fig. 6). In detail, LACE 2.0 returns: (a) the longitudinal clonal tree (generated via Cytoscape.js [14]), (b) the fishplot (generated via TimeScape [15]), (c) a summary table in which the optimal values of false positive $\alpha$ and false negative $\beta$ rates, the number of selected mutations and the number of single cells are displayed, for each time point. All plots are interactive and the properties of the distinct clones at any time are shown via mouse-over (e.g., clonal prevalence, distinctive mutations, etc.). Importantly, the genes hit by the mutations present in any given clone are highlighted and, by selecting their names, LACE 2.0 performs a direct query to the Ensembl gene annotation database [6], so as to facilitate any downstream analyses.

**Table 1:** Names, default values, and validity range of the interface parameters for a given mean mapped read depth *C*

Field name	Value	Range	Variation
Alternate counts (field 3.1)	2	$1 < x \ll C/2$	Higher values select events with more supporting ALT reads and remove PCR errors, but bias the results toward high CN/expressed genes.
MAF (field 3.2)	0.01	$0 \le x < 0.5$	Smaller values imply more rare events compared to the reference population.
Variant Frequency (field 3.3)	0.01	$0 < x \le 1$	Smaller values include less common mutations among cells in the experiment.
Minimum depth (field 4.1)	3	$0 \le x \ll C$	Bigger values imply more NA sites per time point.
Max missing value (field 4.2)	0.4	$0\le x\le 1$	Higher values keep mutational sites for which larger number of cells has an NA value.
Minimum median depth (field 4.3)	8	$1\le x \lesssim C$	Bigger values keep only loci with bigger median coverage among cells.
Minimum alt median depth (field 4.4)	4	$1\le x \lesssim C$	Bigger values keep only mutations with more supporting reads in the cell population.
Learning rate (field 5.1)	1		Higher values permit to avoid local minima, but are less accurate.
False positive rates (field 5.2)		$0<x<1$	Lower values imply more constraining data values.
False negative rates (field 5.3)		$0<x<1$	Lower values imply more constraining data values.
MCMC iterations (field 4.4)	10000	$x>1$	
Number of restart (field 5.5)	50	$x\ge 1$	
Early stopping (field 5.6)	500	$x>1$

**Table 2:** Variant calling types of annotation supported by the back-end [9]

Annotation	Explanation
Frameshift insertion	An insertion of one or more nucleotides that cause frameshift changes in protein coding sequence
Frameshift deletion	A deletion of one or more nucleotides that cause frameshift changes in protein coding sequence
Frameshift block substitution	A block substitution of one or more nucleotides that cause frameshift changes in protein coding sequence
Stopgain	A nonsynonymous SNV, frameshift insertion/deletion, nonframeshift insertion/deletion or block substitution that lead to the immediate creation of stop codon at the variant site. For frameshift mutations, the creation of stop codon downstream of the variant will not be counted as “stopgain”!
Stoploss	A nonsynonymous SNV, frameshift insertion/deletion, nonframeshift insertion/deletion or block substitution that lead to the immediate elimination of stop codon at the variant site
Nonframeshift insertion	An insertion of 3 or multiples of 3 nucleotides that do not cause frameshift changes in protein coding sequence
Nonframeshift deletion	A deletion of 3 or multiples of 3 nucleotides that do not cause frameshift changes in protein coding sequence
Nonframeshift block substitution	A block substitution of one or more nucleotides that do not cause frameshift changes in protein coding sequence
Nonsynonymous SNV	A single nucleotide change that cause an amino acid change
Synonymous SNV	A single nucleotide change that does not cause an amino acid change
Unknown	Unknown function (due to various errors in the gene structure definition in the database file)

### LACE 2.0 constraints and computational performance

The purpose of LACE 2.0 is to retrieve from data the most likely longitudinal clonal tree where the genotypes and their respective changes in prevalences during longitudinal experiments are due to driver mutations. Many of the filters proposed in the app have the scope to limit the number of non-driver mutational events passed to the clonal inference process. Indeed, passenger mutations, even though relevant for phylogenetic purposes, tend to increase the number of clones which can cause overfitting, and do not necessarily explain fitness variations. LACE 2.0 allows the user to reduce the number of putative passenger mutations which are less indicative of prevalence variations. It is worth noting that passenger mutations, under the ISA, will tend to clusterize affecting only partially the bifurcation and the main structure of the clonal tree. Another example is synonymous mutations in exonic regions which may induce selective advantage relative to codon usage bias or play functional roles in splicing and folding of mRNA or protein folding. Whether the functional effects of these mutations, mostly related to the quantitative production of proteins, have driver roles in the experiment or evolution of diseases, it is parametrically decided by the user.

During variant calling, germline mutations should be filtered using a WT/normal reference, when available. If it is not possible, mutations with sufficiently low values of MAF can be selected during the LACE 2.0 pipeline to reduce common polymorphisms and germline mutations.

Filtering mutational events based on the minimum number of reads would imply neglecting possible noisy sequencing effects as PCR duplication errors or selecting events with a more supporting evidence signal. Nevertheless, some cautions are required to avoid results biased toward regions characterized by higher copy number or overexpression by choosing the minimum number of reads well below half the mean mapped read depth.

Further filters on the rate of NA values or depth are computed to the whole set of cells per time point and are used to further select fewer relevant mutational events.

LACE 2.0 is composed by a frontend part which allows to manage projects, filters and displays graphical output and by a backend part which executes the pipeline steps and the sanity checks.

Because the computational time is interleaved by user interactions with the app, the runtime measurements were split in three major lapses: annotation, depth computation (see Table 3) and clonal inference (see Table 4). Each run was repeated 15 times and averaged values per number of cells and number of mutations was reported. The tests were performed using 1000 MCMC iterations and 5 restarts on an

Intel(R) Xeon(R) Gold 6240 2.60 GHz Linux machine with 1 TB of RAM.

**Table 3:** Average runtimes over 15 times repetitions for different numbers of cells and numbers of mutations in seconds for annotation and depth computation

# cells	Annotation	Depth comp
20	2.35	64.59
40	4.38	111.96
60	6.51	163.75

**Table 4:** Inference average runtimes over 15 times repetitions for different sizes of the data matrix $\tilde{D}$

$\tilde{D}(\#\ \textrm{cells},\#\ \textrm{mutations})$	5	10	15
1200	3.1	6.4	25.3

$\tilde{D}(\#\ \textrm{cells},\#\ \textrm{mutations})$	15		
120	4.2		
600	13.8		
1200	25.3		

## Discussion

Former releases of LACE do not use standard VCF and BAM formats as input of the clonal analyses. LACE [4] input was a list of samples for each time point such that an element is a matrix of dimension given by the number of cells pooled in the sample times with all the mutations observed and entries equal to 1, 0 or NA for observed, non-observed mutations or insufficient reads cases, respectively. On the other hand, LACE 2.0 allows the user to infer the SC longitudinal clonal tree starting from largely available formats which are outputs of many SC gold standard alignment and variant calling pipelines. Formerly, the derivation of putative drivers and relevant mutations was left out from the important inferential step, and users needed to adopt external independent procedures. Similarly, assignment of mutations to cells as noisy values were derived in pre-quality check routines. In the present release, starting from variant annotations and depth computation, the user can adopt various filters and selection thresholds in order to set SC mutations with low number of reads as not available data and discriminate significant mutations for the understudied longitudinal mutational process.

The output of LACE was the cell attachment matrix and the clonal tree for the longitudinal experiment represented by zeros whether clonal prevalences are null or 1 otherwise. This could have generated some problems in the reconstruction of a mutational tree with the relation imposed by the time constraints of the experiment.

LACE 2.0 returns an augmented longitudinal clonal tree comprehensive of the time tagging and edges which takes into account the time order of the samples, hence correcting for possible misrepresentations of clones’ first appearance when clonal prevalences are zero.

## Conclusions

In this work, we have presented the LACE 2.0 R package for the inference and interactive visualization of cancer evolution models from longitudinal single-cell sequencing experiments. This tool provides an important step in the direction of a wider diffusion of scalable and easy-to-use methods for single-cell analyses in cancer research, especially thanks to its graphical interface and the extremely limited programming skills required to perform any analysis. In fact, in a few well-defined data processing steps, it is possible to generate a high-resolution picture of the evolutionary history of a tumor and, most of all, explicitly assess the impact of any given therapy. Overall, we advocate the use of LACE 2.0 with already existing and widely available SC RNA-seq datasets of patient-derived models, so as to naturally integrate current analyses on gene expression profiles with those on the underlying clonal evolution.

## Data Availability

LACE 2.0 is available at: github.com/BIMIB-DISCo/LACE. The RNA SMARTseq2 accession number for the Melanoma dataset analysed during the current study is GEO: GSE116237. *Operating system(s)*: Linux, Windows, macOS and platform independent via docker. *Programming language*: R. *Other requirements*: R>= 4.2, Samtools Suite, Annovar. *License*: GPL-3. *Any restrictions to use by non-academics*: None

## Abbreviations

SC: Single cells
SNVs: Single nucleotide variants
ISA: Infinite allele assumptios
BAM: Binary alignment map
VCF: Variant call format
PCR: Polymerase chain reaction
